# Serum Cystatin C Levels Are Associated With Obesity in Adolescents Aged 14–17 Years

**DOI:** 10.3389/fendo.2022.816201

**Published:** 2022-02-04

**Authors:** Ying-Xiang Huo, Wei Wei, Yang Liu, Ya-Nan Ma, Jun-Min Tao, Ning-Ning Wang, Xiao-Feng Li, Xin Chen

**Affiliations:** ^1^ Department of Epidemiology, School of Public Health, Dalian Medical University, Dalian, China; ^2^ Department of Neurosurgery, Affiliated Dalian Municipal Central Hospital, Dalian Medical University, Dalian, China; ^3^ Institute of Health Science, China Medical University, Shenyang, China; ^4^ Department of Biostatistics and Epidemiology, School of Public Health, China Medical University, Shenyang, China; ^5^ Department of Food Nutrition and Safety, School of Public Health, Dalian Medical University, Dalian, China

**Keywords:** cystatin C, obesity, adolescent, boys, girls

## Abstract

**Background:**

The association between serum cystatin C levels and obesity has not been fully explored in adolescents. This study aimed to explore the association between serum cystatin C levels and obesity in adolescents of different sexes.

**Methods:**

We conducted a cross-sectional study including 481 adolescents aged 14−17 years. Cystatin C level was measured by immunoassay. Health examinations data, biochemical parameters, and questionnaire information were collected. The restricted cubic spline model analyzed the association between cystatin C levels and obesity in boys and girls.

**Results:**

Boys exhibited significantly higher cystatin C levels than girls, with a mean level of 0.97 ± 0.10 mg/L in boys and 0.86 ± 0.09 mg/L in girls (*P* < 0.001). The restricted cubic spline model suggested that low or high cystatin C levels were associated with an increased risk of obesity in boys, whereas only higher cystatin C levels were associated with an increased risk of obesity in girls.

**Conclusions:**

A U-shaped correlation was observed between serum cystatin C levels and the risk of obesity in boys. However, in girls, the risk of obesity showed a trend of initially increase and then decrease with increasing cystatin C levels. Longitudinal studies should be conducted to further investigate the diagnostic potential of cystatin C in the progression of early obesity in adolescents of different sexes.

## Introduction

Overweight and obesity pose a great threat to population health in various countries. Obesity is one of the significant risk factors for many noncommunicable diseases, including hypertension, dyslipidemia, and some types of cancer (1). However, a large proportion of obesity in adults stems from obesity in childhood. Weight gain during childhood and adolescence is likely to contribute to overweight and obesity for life (2). Meanwhile, overweight or obesity in childhood and adolescence can significantly increase the incidence of adult obesity, as well as obesity-related disease morbidity and early mortality (3). The morbidity rate of obesity in children increased from 4% in 1975 to more than 18% in 2016 (4). A total of 50 million girls and 74 million boys developed obesity worldwide in 2016 (5). More than 50% of adults and nearly 20% of children and adolescents developed overweight or obesity in 2019, according to the latest national data from China (6). Obesity in children also leads to many health problems, including hypertension, high total cholesterol, and impaired glucose tolerance (7). Obesity in children has become a serious public health issue in various countries around the world (8). Therefore, early detection and prompt treatment of children and adolescents with obesity are very important to avoid or delay the occurrence of obesity and chronic diseases in adults.

Cystatin C is an essential protein that is non-glycosylated and filtered by the glomerulus (9). It is considered as an indicator for evaluating renal function (10). Data suggest a potential genetic link between nonalcoholic fatty liver disease and chronic kidney disease in children (11). Studies on adults have reported a positive correlation between cystatin C level and body mass index (BMI) or waist circumference (WC) (12). Further, some data suggested that increased cystatin C levels could be regarded as an early prognostic indicator of vascular risk in children with obesity (13). Many studies have been conducted on cystatin C levels in adults, but few on the association between cystatin C level and overweight or obesity in adolescents. Currently, no normal reference value range exists for cystatin C levels in adolescents. Thus, the primary goal of this cross-sectional study was to investigate the association between cystatin C and overweight or obesity in adolescence. It used the serum cystatin C level as an indicator of early obesity in adolescents to promote early preventive intervention for overweight or obesity so as to reduce the incidence of obesity and obesity-related diseases in adults.

## Materials and Methods

### Study Population

In this study, all participants were selected by random cluster sampling from a high school in Huanggu District, Shenyang City, Liaoning Province. A cross-sectional survey was conducted on health examinations, biological sample collection, and questionnaire administered to these adolescents. Thus, 481 adolescents whose ages ranged from 14 to 17 years were included.

The inclusion criteria were adolescents with complete anthropometric and biospecimen data. We excluded adolescents with kidney disease, endocrine disease, autoimmune disease, and infectious disease as well as those taking any medication by asking the medical history. Furthermore, we identified and excluded one isolated extreme value of cystatin C level (0.39 mg/L).

### Health Examinations

Trained investigators performed measurements under standard conditions and uniform specifications. Anthropometric measurements were recorded using a fully automated electronic scale (equipment and methods were referenced to GB/T26343), and the instruments used were uniform and calibrated.

Height and weight were measured with the adolescents without shoes and wearing light clothes. Height was measured with the adolescents standing on the bottom plate of the human body altimeter. Weight was measured with the adolescents standing on at the center of the weighing scale. The readings were recorded in “cm” and “kg” and recorded to one decimal place. The BMI was equal to weight divided by height squared (kg/m^2^), retained to one decimal place. The WC was measured with the adolescents standing naturally and with a nonelastic tape around the central level of the umbilicus from the end of exhalation. It was recorded in “cm” and read to one decimal place. The waist-to-height ratio (WHtR) was equal to WC (cm) divided by height (cm), without unit.

The blood pressure was measured using a Riva-Rocci sphygmomanometer. An L-type (32−42 cm) or XL type (42−50 cm) cuff model was used according to the upper arm circumference of children. The study participants should not engage in any strenuous exercise within 1 h before the measurement. The bladder was emptied and the participants sat still for more than 10 min before measuring blood pressure. The blood pressure was measured three consecutive times for each adolescent, with no less than 30-s intervals between each measurement. The values were averaged after removing outliers and recorded as mmHg.

### Biological Sample Collection

The blood samples were collected from all adolescents on an early morning fast. The biochemical measurement analyses were performed by the Department of Laboratory Medicine of the Shengjing Hospital Affiliated to China Medical University. The cystatin C level was analyzed with an automated particle-enhanced turbidity immunoassay using the Architect I16200 automated analyzer (Architect, Shandong, China). In addition, fasting plasma glucose (FPG), triglycerides (TG), uric acid (UA), high-density lipoprotein (HDL), low-density lipoprotein (LDL), and total cholesterol (TC) were measured with a Hitachi 7600 clinical analyzer (Hitachi, Tokyo, Japan). Calculate estimated glomerular filtration rate (eGFR) using the eGFR = k * patient’s length (cm)/serum creatinine (mg/dL) formula (14).

### Questionnaire Administered

The surveys were conducted during face-to-face interviews at the school by trained interviewers. Structured questionnaire was administered to collect data on demographic information (name, age, sex, ethnicity, address, and number of family members), daily dietary intake (eating attitudes, dietary control, eating behaviors, and food preferences), and physical activity information (daily activities and sports activities within 7 days).

Approval was obtained from the ethics committee of Dalian Medical University. Informed consent was obtained from all adolescent and their parents.

### Definitions of Obesity

The BMI of participants was defined as normal, overweight, or obesity according to the Chinese reference norms for overweight and obesity in children and adolescents (15).

Children and adolescents with abdominal obesity were identified using the WC ≥90th percentile value for age and sex according to the standard definition of overweight and obesity in children worldwide by the International Obesity Working Group (16).

The value of WHtR ≥0.5 was suggested to determine central obesity for children (17).

Hypertension was defined as a mean systolic blood pressure (SBP) and/or diastolic blood pressure (DBP) at ≥95th percentile for age, sex, and height on ≥3 times (18).

### Statistical Analysis

Mean and standard deviations were used to represent normal distributions for continuous variables. Medians and interquartile ranges were used to define skewed distributions. In contrast, categorical variables were described as counts and percentages. Adolescents were analyzed stratified by sex. The means of cystatin C levels in groups with obesity and groups without obesity defined by the three criteria (BMI, WC, and WHtR) were analyzed using the *t-*test. The factors independently associated with obesity were identified by univariate logistic regression. Trend tests for cystatin C quartile levels with obesity risk were also conducted, resulting in odds ratio (OR) and 95% confidence interval (CI). ORs at other levels were calculated with binary logistic regression using the lowest quartile of cystatin C as a reference. We used restricted cubic spline (RCS) models fitted for linear regression models and logistic regression models. Knots were present at 10th, 50th, and 90th percentiles, the reference cystatin C level of the logistic regression model was the median. The RCS based on the linear regression model was used to model the association of cystatin C level and BMI-for-age Z-score (19).

Differences were considered statistically significant when the *P* value was less than 0.05. Statistical analysis was performed on R studio version 3.6.1 software (Copyright 2007 Free Software Foundation).

## Results

### General Information

A total of 481 participants, 199 boys (41.4%) and 282 girls (58.6%), were included, aged between 14 and 17 years. The cystatin C level obeyed a normal distribution with the mean levels in all adolescents being 0.90 ± 0.11 mg/L (**Table 1**). Boys exhibited significantly higher levels of cystatin C level than girls, with a mean level of 0.97 ± 0.10 mg/L in boys and 0.86 ± 0.09 mg/L in girls (*P*<0.001). The data showed that age was not significantly different (*P* = 0.330).

**Table 1:** Distribution of cystatin C levels in different sex and age.

Variable	Category	N (%)	CysC (mg/L)		Statistic	P
			Mean	SD		
total	―	481	0.904	0.110	―	―
sex	boys	199 (41.4)	0.965	0.104	t = 11.787	<0.001
	girls	282 (58.6)	0.860	0.091		
age	14	13 (2.7)	0.875	0.079	F = 1.145	0.330
	15	142 (29.5)	0.916	0.112		
	16	247 (51.4)	0.901	0.104		
	17	79 (16.4)	0.894	0.124		

CysC, cystatin C; SD, standard deviation.

As shown in **Table 2**, we defined obesity using three criteria (BMI, WC, and WHtR). We analyzed the differences in cystatin C levels between adolescents with obesity and adolescents without obesity of different sexes. The prevalence was 30.6% for overweight/obesity defined by BMI, 21.7% for abdominal obesity defined by the WC value, and 31.6% for obesity defined by the WHtR ≥0.5. In obesity defined by the BMI, higher cystatin C levels were observed in adolescents with obesity (*P* = 0.005). Girls with obesity had higher cystatin C levels compared with girls with normal BMI (*P* = 0.003). In contrast, the difference was not significant in boys (*P* = 0.703). However, in both abdominal obesity defined by the WC and obesity defined by the WHtR ≥0.5, no statistically significant differences in cystatin C levels were found between boys with obesity and boys without obesity or between girls with obesity and girls without obesity.

**Table 2:** Association between cystatin C levels and obesity as defined by three criteria: BMI, WC, and WHtR.

Variable	Category	Total					Boys					Girls				
		N (%)	CysC (mg/L)		Statistic	P	N (%)	CysC (mg/L)		Statistic	P	N (%)	CysC (mg/L)		Statistic	P
			Mean	SD				Mean	SD				Mean	SD		
BMI	Normal	334 (69.4)	0.894	0.110	t= -2.823	0.005	129 (64.8)	0.963	0.098	t= -0.382	0.703	205 (72.7)	0.851	0.094	t= -2.991	0.003
	overweight/obesity	147 (30.6)	0.925	0.106			70 (35.2)	0.969	0.115			77 (27.3)	0.884	0.078		
WC	Normal	372 (78.3)	0.904	0.106	t=0.270	0.787	165 (83.8)	0.963	0.096	t= -0.363	0.719	207 (74.5)	0.858	0.089	t= -0.888	0.377
	obesity	103 (21.7)	0.901	0.118			32 (16.2)	0.972	0.137			71 (25.5)	0.869	0.093		
WHtR	normal	325 (68.4)	0.901	0.095	t= -0.846	0.398	135 (68.5)	0.964	0.095	t= -0.111	0.912	190 (68.3)	0.856	0.091	t= -1.252	0.212
	obesity	150 (31.6)	0.910	0.120			62 (31.5)	0.966	0.120			88 (31.7)	0.871	0.089		

CysC, cystatin C; SD, standard deviation; BMI, body mass index; WC, waist circumference; WHtR, waist to height ratio.

### Univariate Analysis of Traditional Blood Biochemical Parameters Related to BMI-Defined Obesity

**Table 3** shows the blood biochemical parameters of traditional risk factors for obesity in adolescents stratified by sex. In boys, TG, HDL, LDL, UA, TC, and SBP were found to be associated with the obesity risk in adolescents. However, in girls, age, TG, HDL, UA, and SBP were associated with the obesity risk (*P* < 0.05).

**Table 3:** Univariate analysis of obesity risk factors in boys with obesity and girls with obesity defined by BMI criteria.

Variable	Boys			Girls		
	OR	95% CI	*P*	OR	95% CI	*P*
age	1.142	[0.748, 1.745]	0.539	0.702	[0.495, 0.995]	0.047
TG	3.245	[1.563, 6.738]	0.002	2.623	[1.410, 4.879]	0.002
HDL	0.126	[0.036, 0.445]	0.001	0.302	[0.124, 0.738]	0.009
LDL	3.082	[1.711, 5.553]	<0.001	1.480	[0.957, 2.290]	0.078
FPG	1.277	[0.690, 2.363]	0.436	0.777	[0.356, 1.695]	0.526
UA	1.010	[1.006, 1.015]	<0.001	1.012	[1.007, 1.017]	<0.001
SBP	1.061	[1.035, 1.089]	<0.001	1.064	[1.038, 1.091]	<0.001
DBP	1.013	[0.983, 1.044]	0.385	1.022	[0.994, 1.052]	0.129
TC	1.898	[1.192, 3.021]	0.007	1.296	[0.881, 1.906]	0.188
eGFR	1.008	[0.973, 1.043]	0.660	0.993	[0.972, 1.014]	0.505

OR, odds ratio; 95% CI, 95% confidence interval; TG, triglyceride; HDL, high density lipoprotein; LDL, low density lipoprotein; FPG, fasting plasma glucose; UA, uric acid; SBP, systolic blood pressure; DBP, diastolic blood pressure; TC, total cholesterol; eGFR, estimated glomerular filtration rate.

### Tendency Test of Cystatin C Quartile Levels With Obesity Defined by BMI

**Table 4** presents the associations between cystatin C quartiles and the risk of obesity in adolescents, stratified by sex. Model 1 adjusted for age in different sexes. Model 2 adjusted for age, TG, TC, HDL, LDL, UA, and SBP in boys, but it adjusted for age, TG, HDL, UA, and SBP in girls.

**Table 4:** Analysis of the strength of association between each level of cystatin C quartile by sex and obesity defined by BMI as a criterion in adolescents.

Boys					Girls				
CysC (mg/L)	Model 1		Model 2^a^		CysC (mg/L)	Model 1		Model 2^b^	
	OR (95%CI)	*P*	OR (95%CI)	*P*		OR (95%CI)	*P*	OR (95%CI)	*P*
Quartile 1 (0.67-0.90)	1.00	―	1.00	―	Quartile 1 (0.60-0.80)	1.00	―	1.00	―
Quartile 2 (0.91-0.96)	0.922 (0.397-2.143)	0.850	0.896 (0.336-2.387)	0.826	Quartile 2 (0.81-0.86)	4.374 (1.826-10.480)	0.001	3.847 (1.461-10.134)	0.006
Quartile 3 (0.97-1.04)	1.130 (0.509-2.506)	0.764	1.246 (0.487-3.187)	0.646	Quartile 3 (0.87-0.92)	3.632 (1.459-9.043)	0.006	2.904 (1.058-7.970)	0.038
Quartile 4 (1.05-1.27)	1.570 (0.675-3.653)	0.295	1.377 (0.497-3.817)	0.539	Quartile 4 (0.93-1.10)	3.396 (1.384-8.336)	0.008	2.451 (0.904-6.646)	0.078

Model 1: adjusted for age.

Model 2: a. adjusted for model 1+ TG+ TC+ HDL+ LDL+ UA and SBP.

b. adjusted for model 1+ TG+ HDL+UA and SBP.

CysC, cystatin C; UA, uric acid; TC, total cholesterol; SBP, systolic blood pressure; TG, triglyceride; HDL, high density lipoprotein; LDL, low density lipoprotein; OR, odds ratio; 95% CI, 95% confidence interval.

The boys, had a trend of increasing obesity risk with increasing cystatin C levels. In model 2, taking the cystatin C first quartile level as the reference group, the risk of obesity was reduced by 10% at the second quartile level (OR = 0.90, 95% CI: 0.34–2.39, *P* = 0.826), while it was increased by 25% at the third quartile level (OR = 1.25, 95% CI: 0.49–3.19, *P* = 0.646) and 38% at the fourth quartile level (OR = 1.38, 95% CI: 0.50–3.82, *P* = 0.539) cystatin C levels. However, this growth trend was not statistically significant (*P* > 0.05).

In contrast, in girls, obesity risk showed a decreasing trend with increasing cystatin C levels. In model 2, taking the cystatin C first quartile level as the reference, the risks of obesity were elevated by four-, three-, and two-fold in the second quartile (OR = 3.85, 95% CI: 1.46–8.13, *P* = 0.006), third quartile (OR = 2.90, 95% CI: 1.06–7.97, *P* = 0.038), and fourth quartile (OR = 2.45, 95% CI: 0.90–6.65, *P* = 0.078) cystatin C levels, respectively.

### Dose-Response Association of the Cystatin C Level With Obesity Defined by BMI Based on RCS Models

**Figure 1:**
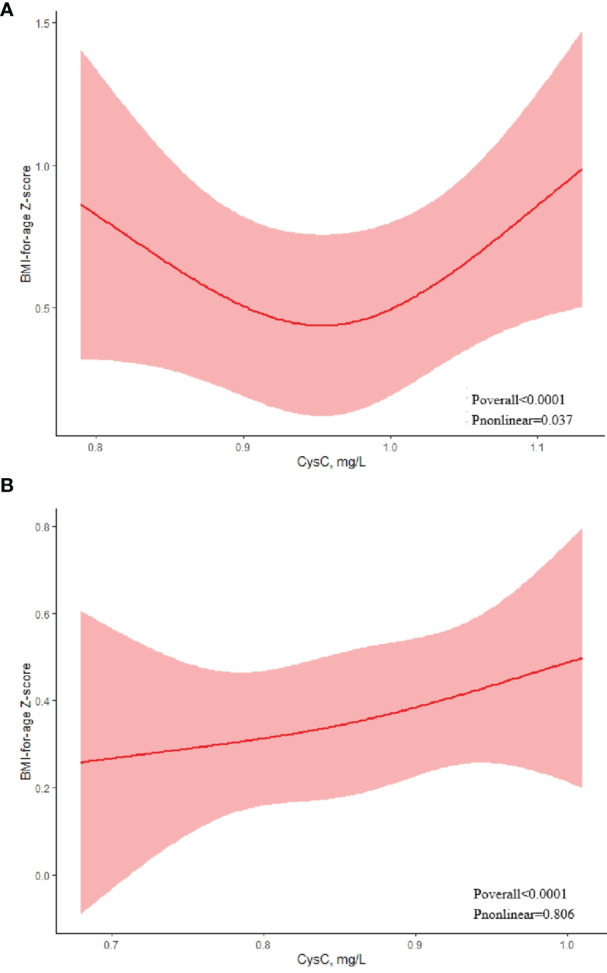
Association between serum cystatin C and BMI-for-age Z-score using a restricted cubic spline regression model in boys **(A)** and girls **(B)**.

We used RCS to explore the dose-response association between cystatin C level and obesity. The association between BMI-of-age Z-score and cystatin C levels on a continuous scale was U-shaped in boys (**Figure 1A**). We estimated the BMI-of-age Z-score to reach a nadir at cystatin C of 0.95mg/L, with inverse associations below and positive associations above (*P*_nonlinear_ =0.037). However, in girls, a positive association was found between BMI-of-age Z-score and cystatin C level (**Figure 1B**). That is, BMI-of-age Z-score gradually increased with increasing cystatin C levels (*P*_nonlinear_ = 0.806).

**Figure 2:**
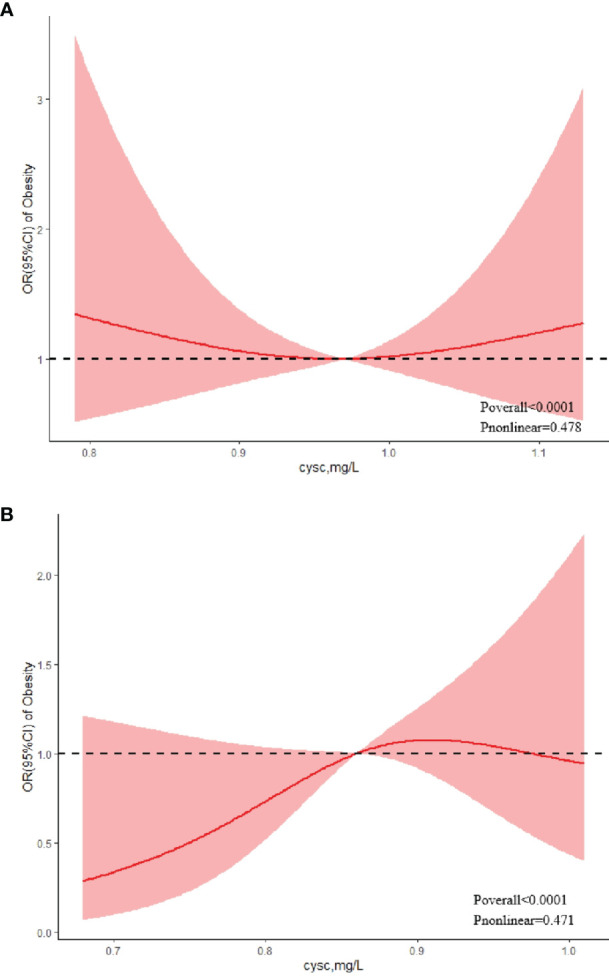
Association between serum cystatin C and risk of obesity using a restricted cubic spline regression model in boys **(A)** and girls **(B)**.

Regarding the strong U-shaped relationship between the risk of obesity and cystatin C levels in boys, the plot showed a decreasing risk trend in the lower range of predicted cystatin C levels, reaching a minimum risk around 0.97 mg/L and then increasing (*P*_nonlinear_ = 0.478). Below 0.97 mg/L, the OR per standard deviation higher predicted cystatin C levels was 1.45 (0.71–1.84). Above 0.97 mg/L, the OR per standard deviation higher predicted cystatin C levels 1.12 (0.70–1.78) (**Figure 2A**). The risk of obesity showed an increasing trend with elevated cystatin C levels before the cystatin C level reached 0.91 mg/L in girls, with the highest risk of obesity (OR = 1.07, 95% CI: 0.88–1.31) at the cystatin C levels of 0.91 mg/L (**Figure 2B**). After that, the risk of obesity tended to decline (*P*_nonlinear_ = 0.471).

## Discussion

The present study proposed that the cystatin C level in adolescents was associated with obesity, particularly obesity defined by BMI. These data suggested that the cystatin C level could be considered as an early biomarker of obesity in adolescents. There was a U-shaped association between cystatin C levels and obesity in boys, and the risk of obesity showed a trend of first decrease and then increase as cystatin C levels increased, and either low or high cystatin levels were associated with an increased risk of obesity. Whereas in girls, the risk of obesity showed a trend of initially increase and then decrease with increasing cystatin C levels.

The study found that boys had higher cystatin C levels than girls, which was consistent with other findings that boys had higher cystatin C levels than girls after 1 year of age (20). The possible reason for the result might be that adolescent boys had a higher increase in muscle mass than girls (20). In addition, renal factors, hormonal factors, or other factors interfering with the determination of the cystatin C level in individual girls might also explain sex-related differences (21). Several findings suggested that individuals began to produce more cystatin C when they were around 10–13 years of age. After the age of 13 years, the individual’s cystatin C production reached a peak. It then remained stable until around the age of 50 years (22). Considering the particularity of children’s growth and development, and that the distribution of cystatin C did differ between boys and girls, we analyzed sex as a stratification factor in the next analysis. About 80% of adolescents with obesity still obese in adulthood, and hence early preventive interventions for obesity are warranted, however, childhood BMI is less sensitive to predict adult obesity (23), so we need to find other more sensitive indicators for detecting obesity in adolescents. Cystatin C is an effective cathepsin-proteolytic enzyme inhibitor (24). It can be used to calculate the eGFR (25). Studies have also shown a significant association between GFR and duration of obesity (26). However, in our study, no relationship between eGFR and obesity was found. The present study showed that the elevated circulating cystatin C levels in study participants with obesity may be to counteract obesity-related inflammation (27). Elevated cystatin C levels in adolescence were also associated with lipid metabolic dysregulation and cardiometabolic risk (28). The production of cystatin C in adipose tissue can lead to an increase in the concentration of cystatin C in the blood of children with obesity (29). The studies demonstrated that cystatin C level was associated with WC, BMI, and visceral fat (30–32). However, our study only found a relationship between BMI-defined obesity and cystatin C levels. It also provided a rationale for using cystatin C as a biomarker to predict overweight and obesity in adolescents.

Our study found a U-shaped association between cystatin C levels and obesity in boys, with a decreasing risk of obesity before cystatin C values reached 0.97 mg/L and then increasing; however, in girls, the risk of obesity increased before the cystatin C value reached 0.91 mg/L and then tended to decrease. The different associations we observed between the cystatin C levels and obesity risk of different sexes were interesting. Since the age distribution of our study population was in adolescence (14 – 17 years), this difference may have resulted from different levels of pubertal development in boys and girls. To our knowledge, no study had found this U-shaped association of cystatin C levels with the risk of obesity in boys. Therefore, we hope that prospective studies with larger sample sizes will be followed to confirm the differences of different cystatin C levels with obesity risk among boys and girls. The typical reference range in adults for Cystatin cystatin C was 0.40–1.10 mg/L (33). However, no explicit reference value range exists for children and adolescents. Whether normal cystatin C values ranges for boys and girls separately need to be developed in the future for early diagnosis of obesity remains to be explored.

This analysis had several limitations. First, the adolescent population we studied was obtained by cluster sampling and is not nationally representative. Second, critical adverse factors, such as pubertal development and family history, as well as insulin resistance levels, were not considered in our analysis due to limited data. Population-based prospective studies should be carried out to overcome these limitations and comprehensively understand the cystatin C levels in adolescents with obesity and adolescents without obesity.

In conclusion, this study suggested a U-shaped association between serum cystatin C levels and obesity in boys, with low or high cystatin C levels both associated with an increased risk of obesity. However, in girls, the risk of obesity showed a trend of initially increase and then decrease with increasing cystatin C levels. Prospective studies with larger sample sizes should be conducted to evaluate the value of cystatin C levels in predicting the progression of early obesity in adolescents of different sexes.

## Data Availability Statement

The raw data supporting the conclusions of this article will be made available by the authors, without undue reservation.

## Ethics Statement

The studies involving human participants were reviewed and approved by Dalian Medical University Ethics Committee. Written informed consent to participate in this study was provided by the participants’ legal guardian/next of kin. Written informed consent was obtained from the minor(s)’ legal guardian/next of kin for the publication of any potentially identifiable images or data included in this article.

## Author Contributions

Y-XH conceived data, wrote the original draft and performed the primary analysis. YL and Y-NM carried out the field data collection. WW and J-MT extracted the data and helped with the analysis. N-NW and X-FL performed the formal analysis. XC provided design ideas, controlled the analytical methods, and edited the review & editing. All authors reviewed and approved the final manuscript. XC acts as guarantor. The corresponding author attests that all listed authors meet authorship criteria and that no others meeting the criteria have been omitted.

## Funding

This work was supported by the National Natural Science Foundation of China (grant numbers 81602871).

## Conflict of Interest

The authors declare that the research was conducted in the absence of any commercial or financial relationships that could be construed as a potential conflict of interest.

## Publisher’s Note

All claims expressed in this article are solely those of the authors and do not necessarily represent those of their affiliated organizations, or those of the publisher, the editors and the reviewers. Any product that may be evaluated in this article, or claim that may be made by its manufacturer, is not guaranteed or endorsed by the publisher.

## Abbreviations

BMI, Body mass index; WC, waist circumference; WHtR, waist-to-height ratio; TG, triglycerides; HDL, high-density lipoprotein; LDL, low-density lipoprotein; TC, total cholesterol; UA, uric acid; FPG, fasting plasma glucose; SBP, systolic blood pressure; DBP, diastolic blood pressure; OR, odds ratio; CI, confidence interval; RCS, restricted cubic spline; eGFR, estimated glomerular filtration rate.
